# A review of inferior vena cava filters

**DOI:** 10.1259/bjr.20211125

**Published:** 2022-08-03

**Authors:** Kevin P. Sheahan, Emma Tong, Michael J. Lee

**Affiliations:** Department of Radiology, Beaumont Hospital, Dublin, Ireland; Department of Radiology, Beaumont Hospital, Dublin, Ireland; Department of Radiology, Beaumont Hospital, Dublin, Ireland

## Abstract

The care of patients with venous thromboembolism (VTE) is delivered via a multidisciplinary team. The primary treatment for VTE is anticoagulation; however, placement of filter devices in the inferior vena cava (IVC) to prevent embolisation of deep venous thrombosis (DVT) is a well-established secondary treatment option. Many controversies remain regarding utilisation and management of filters.

There are two types of IVC filters, non-retrievable (permanent) and retrievable (optional). We reviewed the literature on the type of IVC filters, indications for placement, contemporary guidelines for placement, complications, management and potential future guidance.

Guidelines differ in their recommendations in these clinical scenarios, however, do concur that retrievable IVC filters are indicated in patients with VTE who have an absolute contraindication to anticoagulation. Broader indications for IVC filters have expanded, however, despite no data demonstrating a mortality benefit, IVC filter use has increased consistently.

Unretrieved filters can lead to DVT, filter migration/embolisation, filter fracture, IVC perforation, and filter-related caval thrombosis. Structured follow-up programs increase retrieval rates, and detect and potentially reduce complications. Multidisciplinary pulmonary embolism response teams (PERTs) have been developed, which could reduce unnecessary IVC filter placements, and facilitate follow up in a specific VTE clinic and could lead to higher filter retrieval rates.

## Introduction

Care of patients with venous thromboembolism (VTE) is delivered via a multidisciplinary team and includes medical and interventional management. Anticoagulation is the primary treatment for VTE^1^ ; however, insertion of filter devices in the inferior vena cava (IVC) to prevent embolisation of deep venous thrombosis (DVT) is a well-established alternative treatment option. Many controversies remain regarding the utilisation and management of filters.^2^ There is a paucity of data supporting filter use with few prospective randomised trials. An electronic search of PubMed and MEDLINE was performed for relevant publications, using subject-related keywords to search recent literature within the preceding three years.

A recent systematic review by Liu et al^3^ published in 2021 included 7 articles with 1274 patients. The authors found no significant difference in pulmonary embolism (PE)-related mortality between the IVC filter group and control groups at 3 months (risk difference, −.01; 95% CI, −.03 to 0.00; *p* = .11) including during the whole follow-up period with low heterogeneity (I²=0%). New PE recurrence within 3 months and during the entire follow-up period was lower in the IVC filter group compared to the control group (0.81% vs  5.98%; risk ratio (RR), 0.17; 95% CI, 0.04–0.65; *p* = .01; and 3.2 vs 7.79%; RR, 0.42; 95% CI, 0.25–0.71; *p* = .001, respectively). The authors found no significant differences in rates of a DVT recurrence or mortality during the entire follow-up period between both groups (*p* > .05). IVC filter use has demonstrated no definitive overall mortality benefit, but insertions increased steadily from 1979 to 2010.^4^

## IVC filters

**Figure 1.**
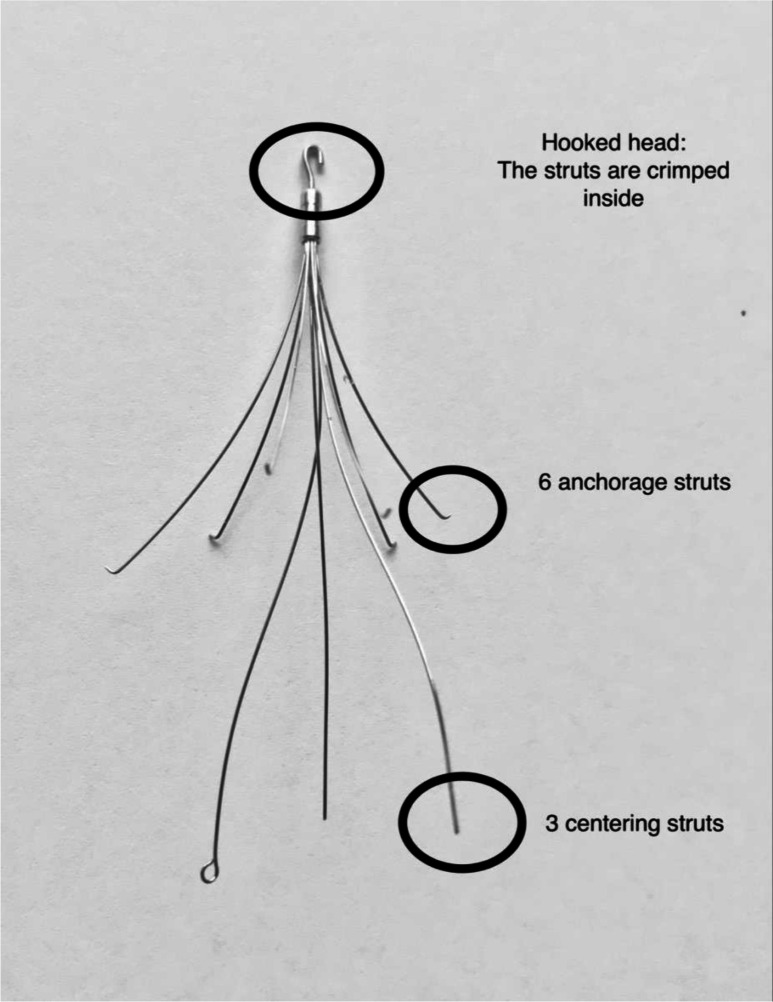
The ALN Filter (Implants Chirurgicaux, France) is our current filter of choice.

Design of absorbable bioconvertible filters is underway. Generally, there are two types of IVC filters, non-retrievable (permanent) and retrievable (optional, *e.g.* ALN filter (*Implants Chirurgicaux, France*), Figure 1). Patients with clear long-term contraindications to anticoagulation and a clinical requirement to prevent PE have permanent filters placed.^5^

Retrievable filters should be removed, when clinically feasible, if contraindications to anticoagulation or risk of PE end. At our institution, we prefer retrievable filters in most cases; however, specific instances, including a cancer diagnosis, or previous failure of anticoagulation, may favour a permanent filter.^6^ No type of retrievable filter is considered more effective than others,^7^ and no specific placement technique is recommended.^8^ However, if retrieved, retrievable filters have the potential advantage of less long-term complications associated with permanent filters, such as the risk of filter migration, subsequent DVT, and IVC stenosis or occlusion.^9^ A systematic review in 2011 of 37 trials, including 11 prospective clinical trials consisting of 6834 patients who had retrievable IVC filters placed, found a PE rate of 1.7% following filter placement, suggesting retrievable filters are as effective in preventing PE if used as permanent filters.^10^ Table 1 demonstrates a variety of filter types.

**Table 1.** Commonly placed IVC filters^11–13^

Manufacturer	Name	MRI compatibility	Permanent/Retrievable?	Approved caval diameter (mm)
ALN	ALN vena cava filter	Conditional to 3T	Yes	32
Argon	OptionElite	Conditional to 3T	Yes	30
Bard	Denali	Conditional to 3T	Yes	28
Bard	Recovery G2	Conditional to 3T	Yes	28
Boston Scientific	Sentry	Conditional to 3T	No (Bioconvertible)	28
Boston Scientific	Greenfield	Safe to 1.5T	No	28
B.Braun	VenaTech LP	Conditional to 3T	No	28
B.Braun	VenaTech	Conditional to 3T	No (Convertible)	28
Cook	Celect	Conditional to 3T	Yes	30
Cook	Gunther Tulip	Conditional to 3T	Yes	30
Cook	Bird’s Nest	Conditional to 3T	No	40
Cordis	OPTEASE	Conditional to 3T	Yes	30

IVC, inferior vena cava.

## Indications and guidelines

The Society of Interventional Radiology (SIR) published current guidelines in 2020 in collaboration with the American College of Cardiology, American College of Chest Physicians, American College of Surgeons Committee on Trauma, American Heart Association, Society for Vascular Surgery (SVS), and Society for Vascular Medicine (SVM).^8^ The expert panel reviewed 34 studies to provide the evidence base for the guidelines and agreed on 18 recommendations, summarised in Table 2.

**Table 2.** Complications following IVC filter insertion^12^

Complication	Rate of occurrence
Filter migration/embolisation (*i.e.* >2 cm change from original position)	<1%^10^ >90% occur after 30 days
Filter fracture	2–10%^14^ 40% risk of fracture at 5.5 years^15^
IVC perforation: filter head or strut >3 mm beyond the wall of the IVC or within an adjacent structure	0–41%^16^ 20% of IVC filter complications reported as IVC perforation^10^
DVT	PREPIC cohort^17^ Incidence at 2 years: 21% Incidence at 8 years: 36% Hazard ratio 1.49 compared with patients without IVC filters
Filter-related thrombosis	2–30%^14^

DVT, deep venous thrombosis; IVC, inferior vena cava.

### Typical indication for IVCF placement

The typical indication for placement of an IVC filter is the existence of VTE with an absolute contraindication to anticoagulation, a complication of anticoagulation, or failure of anticoagulation.^18^ The advantage of IVC filter insertion in the setting of acute PE is the prevention of morbidity and mortality from haemodynamic effects of PE recurrence. However, limited low-quality observational studies support this practice.^19,20^ Routine use of IVC filters is not recommended (Class IIIa evidence) in patients with VTE treated with therapeutic anticoagulation.^8,21^

IVC filter placement should be considered when anticoagulation needs to be stopped due to occurrence of significant bleeding.^22^ A cohort study of patients with significant bleeding risk and VTE demonstrated a lower risk of PE-related mortality in the filter group but a higher rate of VTE recurrence.^22^

A cohort study found filter placement reduced 3-month all-cause mortality in anticoagulated patients who experienced recurrent PE. However, they demonstrated no difference in patients with DVT recurrence, and mortality related to PE was similar.^23^ Many causes for failure of anticoagulation are potentially addressable, including suboptimal medication adherence, drug–drug interactions, inappropriate dosing, or anatomic disorders that predispose to VTE. The SIR recommends filter placement only in patients with objectively confirmed VTE recurrence and no modifiable issue related to anticoagulation therapy.^8^ In any case of failed anticoagulation, if inserting a filter, hypercoagulable conditions, including antiphospholipid antibody or Trousseau syndrome, should be excluded prior to placement to avoid significant morbidity.^24^ In addition, the risk of cardiopulmonary deterioration should outweigh the ongoing thrombotic risk from a filter placement.

### Broader evidence for IVC filter insertion

Broader evidence for IVC filters has expanded, including insertion in addition to anticoagulation in those with VTE recurrence, progression of, or proximal DVT, high-risk PE with coinciding DVT, thrombolysis for Iliocaval DVT, thrombolysis or thrombectomy for large PE, problems with maintenance of anticoagulation, and anticoagulation complications.^1^

A comparative study including 13,125 patients suggested adjuvant filter placement may decrease mortality for patients with acute PE as the authors showed decreased in-hospital mortality to 2.6% from 4.7% when IVC filters were placed in patients with PE, regardless of anticoagulation status.^25^

In the above clinical scenarios, guidelines appear to differ; however, we agree with the placement of retrievable IVC filters for patients who have an absolute contraindication to anticoagulation and VTE,^1,9,21^ despite no evidence demonstrating any definite mortality benefit. Table 2 summarises society guidelines on indications for IVC filter placement.

No large-scale randomised prospective clinical trials have compared outcomes with or without filters in patients with VTE who cannot receive anticoagulation therapy. Randomised studies investigating the potential benefits of IVC filters are more attainable than these trials. Two prospective randomised controlled trials (RCT) evaluated the effectiveness of caval filters in addition to anticoagulation. The initial PREPIC^26^ trial in 1998, a prospective multicentre non-blinded RCT, involved 400 patients with a proximal iliofemoral DVT at high risk for PE. All patients were initially anticoagulated with heparin and subsequently with warfarin. They randomised patients to receive a permanent filter with anticoagulation or anticoagulation alone. At 12 days, they repeated imaging or if suspicion for a new PE transpired. The incidence of PE on Day 12 was the primary end point. Patients who had a filter placed and anticoagulation had a significant reduction in risk for PE at 12 days (1.1% *vs* 4.8%; *p* = 0.03); however, no reduction in PE at 2 years or all-cause mortality and an increased risk of repeat DVT (21% *vs* 12%; *p* = .02). The authors recommend against the routine use of IVC filters.^17^ An additional meta-analysis on the efficacy and safety of vena cava filters included 11 studies has supported these results.^27^ This review had 2055 patients who received a filter *vs* 2149 controls. Filter placement was associated with a 50% decrease in PE incidence and *a* ~70% increase in DVT risk. There was no difference between groups in all-cause mortality or PE-related mortality.

The authors observed the patients in the PREPIC cohort for 8 years.^26^ The 8-year follow-up study reported decreased non-fatal PE occurrence but an increase in delayed recurrent DVT amongst filter recipients (36% *vs* 28%; *p* = .042), and overall survival was not affected. Long-term anticoagulation treatment was similar in both groups, suggesting differences in anticoagulation did not influence the increased DVT rate in the filter group. Recent studies support these findings, which suggest filters reduce the risk of recurrent PE to approximately 1–3% but increase the risk of lower limb DVT.^22,28^

The PREPIC2 RCT, published in 2015, including 399 patients, was conducted following the introduction of temporary IVC filters.^29^ Patients had at least one marker for increased severity, including age >75 years, active cancer, poor cardiac or respiratory reserve, and were in the hospital with acute, symptomatic PE and DVT. All patients were anticoagulated for 6 months; the treatment group had temporary filters inserted, and the authors planned to remove them in 3 months. At 3 and 6 months, the rate of recurrent VTE was low, with no significant differences between the groups, but a tendency towards PE recurrence and higher mortality in the IVC filter group. The authors performed efforts to retrieve filters at 3 months in 91% of the group, of which 93% were successful.^29^ These studies confirmed that there was no indication found for IVC filter placement. Notably, filter efficacy in patients with confirmed PE, DVT, or both who cannot receive anticoagulation treatment (excluding these patients in both trials), was not addressed and, therefore, not representative of typical patients who receive filters.

A recent systematic review and meta-analysis of IVC filters in 2017 included 11 studies with 6 RCTs and 4204 patients.^27^ This review found that IVC filters across various indications are associated with decreased risk of subsequent PE, increased risk of DVT, a non-significant reduced mortality related to PE, but no difference in all-cause mortality.^27^ The same group assessed hospitalisations from 1999 to 2010 in 2016 and found that mortality associated with PE hospitalisations declined regardless of IVC filter use.^30^

While more extensive prospective trials have given insufficient data supporting broader indications for IVC filters, small-scaled studies have examined a more particular patient population who could gain from filter placement. A prospective cohort study of 371 patients with significant bleeding risk and VTE established that the filter group had a lower PE-related death risk (*p* = 0.03) but a high chance for VTE recurrence (*p* =<0.001).^22^ Another small cohort study of patients identified from the RIETE (Registro Informatizado de la Enfermedad Tromboembolica) registry demonstrated patients with recurrent VTE and PE, even with anticoagulation, had a 3 month reduced all-cause mortality rate when inserting an IVC filter. There was no difference in those with recurrent DVT alone (*p* = 0.56), and PE-related mortality was unchanged (*p* = .08).^23^

Studies have shown survival benefits in patients receiving IVC filters with PE who are deemed haemodynamically unstable or critically ill due to mechanical ventilation or shock,^31^ those undergoing pulmonary embolectomy or thrombolysis.^32^ In-hospital survival benefits were also seen with filter placement in unstable elderly patients with acute PE receiving thrombolytic therapy, particularly in patients > 80 years old, who showed the most significant relative risk (RR, 0.35; 95% CI, 0.27–0.46), suggesting that old age should not be a restricing factor when contemplating filter placement for these patients.^33^ These patients may be unable to compensate for even a tiny additional embolisation event and may benefit from filter placement.^34,35^ Patients who have congestive heart failure and PE have an increased mortality rate, and IVC filters are associated with lower all-cause in-hospital mortality.^36^ Compared to patients who did not receive an IVC filter, the authors demonstrate lower mortality rates in retrospective cohort studies for patients in stable condition with PE who received thrombolytic therapy and filter placement.^31,37^

The SIR recommends a multidisciplinary approach regarding filter placement for patients with PE in unstable conditions and other advanced therapies with the benefits of reduced hospital mortality in select patients favouring filter placement.^8^ It is unlikely that we will see large RCTs in these clinical scenarios.

### VTE associated with cancer

Cancer, being a pre-thrombotic state, has a higher probability of VTE, accompanying increased morbidity and mortality. Some authors consider malignancy a contraindication to filter placement, and there is a lack of data supporting filter placement in this population.^38^ While some studies suggest specific subsets of cancer patients with PE may benefit from filter placement,^39,40^ currently, anticoagulation is preferred, with indications for filter placement the same as in the general population.^41^ A sizeable database study (*n* = 14,000) reported that 19.6% of patients with cancer-associated VTE received IVC filters, but only 21% had a documented contraindication to anticoagulation. In a small moderate-quality,^41^ randomised prospective study including 64 patients with VTE associated with malignancy, no benefit was found for additional filter insertion with anticoagulation *vs* anticoagulation alone at 3 month follow-up. There was no reduction in 30 day mortality or recurrent PE at 180 days and an increased risk of DVT at 180 days in the filter recipients.^42^

### Pregnancy and VTE

Pregnancy is a hypercoagulable state, and VTE can complicate 0.5 to 1% of pregnancies. However, the authors excluded pregnant patients in the RCTs of PREPIC and PREPIC2. Anticoagulation with heparin is the suggested treatment for VTE in pregnancy (warfarin is teratogenic), although this may require cessation in patients with high bleeding risk in the peripartum period. Indications for IVC filter placement are the same as in the general population; however, in patients with recent proximal DVT and those with significant PE, retrievable IVC filters may be placed for short periods. A systematic review in 2016 of 44 case reports and case series found that IVC filters may be inserted successfully in selected cases in the prevention of PE in pregnancy.^43^

### Prophylactic IVC filter placement in trauma and bariatric surgery

Prophylactic retrievable filters should be used selectively in trauma and surgical patients who are at high risk of VTE and cannot be treated with anticoagulation prophylaxis.^44^ Trauma patients can be at increased risk for VTE, occurring in up to 50% who do not receive prophylactic anticoagulation due to immobility, endothelial injury, and hypercoagulability; however, IVC filter use remains controversial.^45^ The incidence of filter placement in trauma patients has been reported between 0.6 and 9.6%, with practice varying widely between institutions.^46^ Retrospective studies have reported reduced symptomatic and fatal PE with prophylactic filter insertion in high-risk trauma patients.^47,48^

A multicentre RCT demonstrated no mortality benefit or reduction in symptomatic PE in a group of trauma patients with a contraindication to anticoagulation who received a prophylactic filter within 72 h of presentation compared with a control group. Interestingly, patients who survived at least 7 days and were unable to commence prophylactic anticoagulation by Day 7 had a 14.7% incidence of PE in the control group *vs* no PEs in the treatment group.^44^ However, the authors found no significant difference (RR, 0.00, 95% CI, 0.00–0.55). This finding suggests that trauma patients who cannot be anticoagulated for a prolonged period may benefit from an IVC filter insertion; however, not every trauma patient with an early contraindication to prophylaxis should receive a filter.^49^

A meta-analysis of eight studies demonstrated a reduced PE incidence and reduced PE-related mortality when filters were placed in trauma patients prophylactically, but there was no reduction in DVT or overall mortality.^50^ Stein et al^51^ found an increased PE rates (14.7%) in trauma patients with fractures who had an IVC filter inserted than in those without a filter (0.5%). Other studies have reported no benefit and increased occurrence of DVT.^19,46^

Professional societies have recommended prophylactic filter placement in certain trauma patients at high-risk, particularly those who cannot receive VTE prophylaxis, as there has been no mortality improvement with filter placement which is associated with an increased rate of DVT.^1,8,9,18^

A systematic review of 18 studies of bariatric surgery demonstrated no clear benefit for prophylactic filter placement; however, a small subset of patients with multiple risk factors for VTE had reduced PE-related mortality.^52^ In another subset of patients with PE following joint replacements, adjuvant IVC filter placement has demonstrated fewer complications and overall hospital costs.^53^

### IVC filter placement during endovenous procedures

The FILTER-PEVI trial addressed the issue of filter use during endovenous procedures.^54^ This trial included 141 randomised patients undergoing endovenous intervention for symptomatic DVT who were to receive (*n* = 70) or not receive a retrievable filter (*n* = 71). There was PE detected in 1 of 14 patients with symptoms suggestive of PE in the IVC filter group and 8 of 22 patients in the group without a filter (1.4% *vs* 11.3% of the total population; *p* = .048).^54^ A retrospective cohort study found no difference in rates of PE occurrence or complications when comparing patients undergoing catheter-directed thrombolysis or thrombolysis with and without an IVC filter.^55^

### Free-floating iliofemoral or IVC thrombus

A prospective study demonstrated no increased risk of PE in cases of free-floating thrombus.^56^ There has been no study which has shown better outcomes with IVC filters in place of or in addition to anticoagulation in cases of free-floating iliofemoral or IVC thrombus; however, these cases are still regarded as a relative indication in previous consensus statements.^57^

### Inferior vena cava filter placement^58^

Design of IVC filters with different delivery systems are for either the jugular or femoral approach, so operators need to ensure they are using the appropriate device for the appropriate approach. It is essential to check that the operator has the appropriate device as the Bird’s Nest^®^ Filter (Cook Medical, Bloomington, IN) (Table 1) is currently the only filter for insertion in a megacava.

Following ultrasound-guided access into the right internal jugular vein or femoral vein, place an appropriate catheter in the IVC, and perform venography;

- To demonstrate the patency of the IVC, assess its size and any angulation.
- To confirm conventional anatomy, *i.e.* single IVC.
- To document the position of the renal veins

Use the patient’s spine as a reference for the position of the renal veins and to measure the IVC (a radio-opaque ruler can be placed to the left of the spine if angiography equipment does not include measurement software).

Position the filter below the renal veins ideally. Deployment of each type of filter is different, so read the instructions carefully before using or request help.

## Retrieval

**Figure 2.**
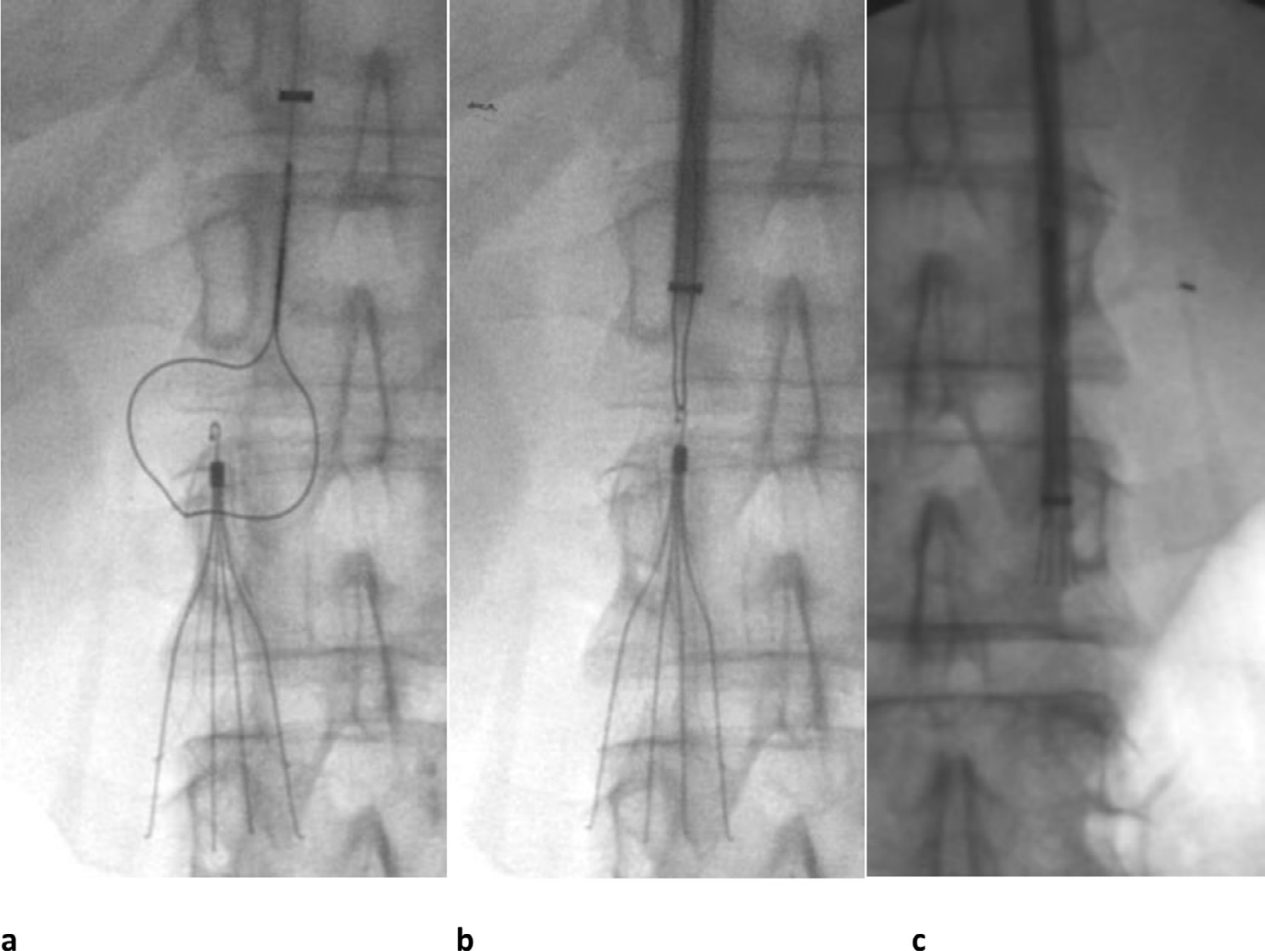
Celect® (Cook) IVC filter removed with retrieval snare set in a 31-year-old male with a history of PE, testicular cancer and retroperitoneal lymph node dissection. (a) After first performing a venogram to ensure no thrombus is present in the filter, the looped snare is manipulated down over the hook. (b) The snare has been tightened around the hook and (c) the sheath is pushed down over the filter while holding the snared filter firmly until the filter is within the sheath. The filter is then removed through the sheath. IVC, inferior vena cava; PE, pulmonary embolism.

**Figure 3.**
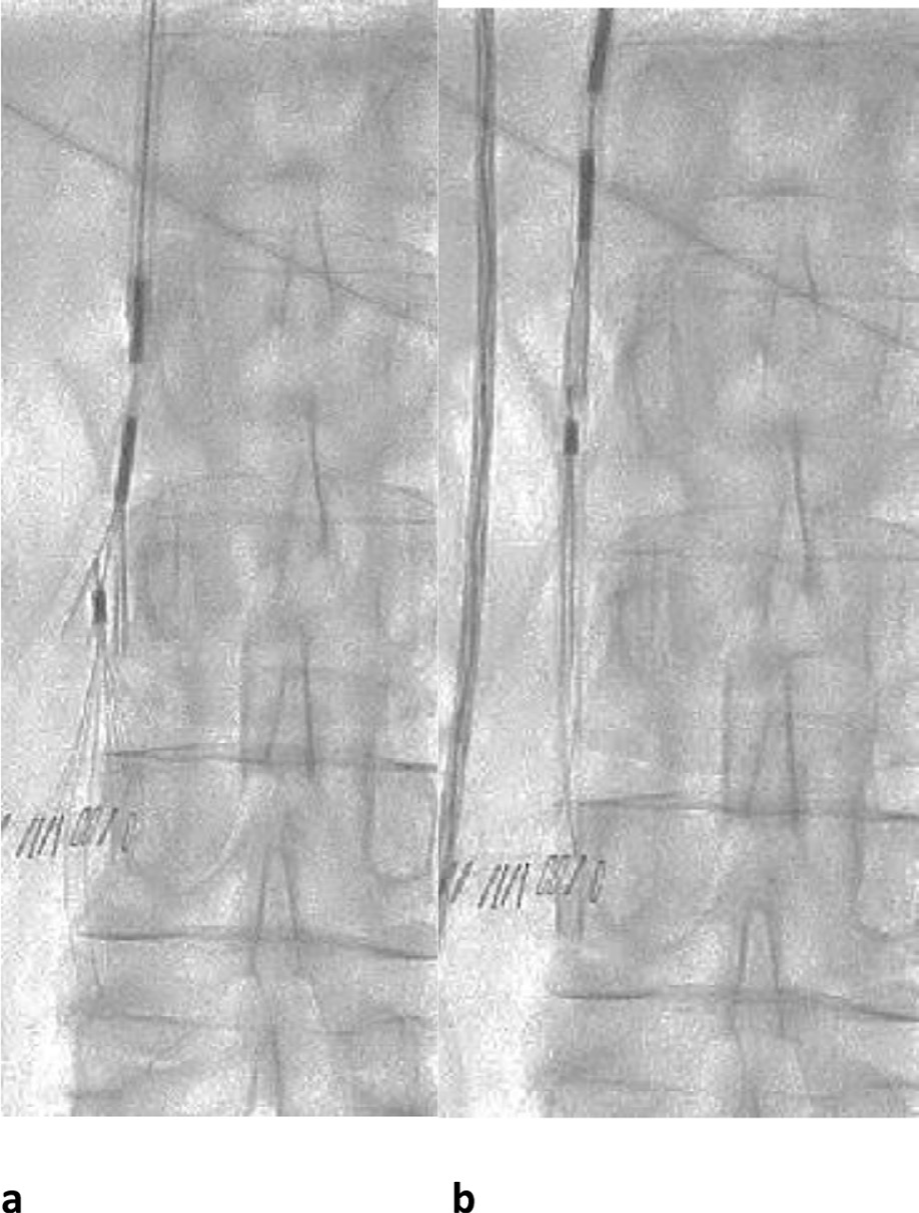
ALN IVC filter retrieval in a 77-year-old lady who had a DVT and needed interruption of anticoagulation for a neurosurgical procedure. (a) 6 weeks after the surgery, the ALN retrieval device was used for retrieval (grasping device with curve on the end of the delivery sheath to facilitate removal of filters tips close to the caval wall). The grasper of the retrieval device is seen directed over the hook of the filter. (b) Once the grasping device has engaged the filter tip, it is closed around it and the sheath advanced over the filter, which is then withdrawn. IVC, inferior vena cava; DVT, deep venous thrombosis.

Retrievable filters inserted with an intent to retrieve should be retrieved when the risk of PE subsides. In 2010, an FDA advisory committee raised concerns over filter complication rates, prompted by a retrospective study by Nicholson et al,^59^ which found increased strut fractures and complications in retrievable filters. They did not make any recommendations against filters or comment on filter indications. In 2010, IVC filter placement peaked in the United States, with 412 filters placed per 100,000 hospitalisations.^4^ By 2014, IVC filter use had reduced to 321.8/100,000 hospitalisations following the FDA’s recommended removal of filters as soon as protection from PE is no longer needed, ideally within 29–54 days after implantation.^60,61^ The complications seen in the Nicholson study did not apply to all models of retrievable filters.^9^ Standard retrieval sets include the ALN grasper or Celect^®^ (Cook) snare loop retrieval kits from a jugular approach (Figures 2 and 3).

## Structured follow-up

More significant efforts should be employed to remove filters as studies have found suboptimal retrieval rates, with only approximately one-third of filters being retrieved.^10,62,63^ O'Keefe et al^64^ found retrieval rates to be higher (55%) in a cohort of trauma patients who had a formal follow-up with a nurse using a tracking system than in those with no documented follow-up (19%). Studies have^65,66^ shown that dedicated IVC filter clinics improved retrieval rates. Automated reminder systems, including electronic tracking of patients and e-mail reminders to plan filter removals, improved rates from 37 to 85%.^67^ Sutphin et al^68^ used an automated outpatient scheduling system to track patients, which led to a 52% increase in retrieval rates. Many institutions report improved retrieval rates by establishing a multidisciplinary team, patient education, a dedicated filter registry, and rigorous follow-up or tracking of filter recipients by a dedicated staff member.^69–71^ The SIR recommends using a structured follow-up program to increase retrieval rates and detect and potentially reduce complications.^8^ Our centre evaluated retrieval rates over nine years following the introduction of a filter registry. Overall retrieval rates were 92%. The mean dwell time was 59 days, with 85% removed within 100 days, highlighting the merit of recordning a prospective IVC filter registry.^72^

Successful retrieval of filters is normally performed within the first 3 months following placement.^10^ A systematic review reported a 94.5% successful retrieval rate in 1815 cases. The most common causes for failed filter retrieval were longer dwell time, increased transverse filter tilt, adherence of the filter hook in the caval wall, and significant thrombus burden, defined as 25% of the filter volume.^10,73^ In 628 retrievals reported in the Cardiovascular and Interventional Radiological Society of Europe (CIRSE) registry,^71^ the average dwell time for successful retrieval was 85 *vs* 145 days for unsuccessful retrievals. A single-centre retrospective review reported a successful retrieval rate of 84.4% in 295 cases over 8 years. The median time for successfully removed filters was 196 days *vs* 375 days for failed retrievals. In this study, 13 attempted permanent filter retrievals had a median dwell time of 3605 days and a 61.5% success rate.^74^ No complications were reported with filter retrievals, suggesting that retrieval should be performed in an expert centre. However, the SIR recommends against the routine removal of permanent filters in patients who are no longer at risk of PE.^8^

Prior to filter retrieval, imaging of the IVC is performed, in most centres, at the time of filter retrieval. CT, MRI, or ultrasound can image the IVC prior to retrieval,^57^ mainly if there are symptoms related to filter complications such as leg swelling or abdominal pain; however, the SIR does not recommend routine pre-procedural imaging.^8^ We perform a pre-retrieval assessment for filter-associated thrombus and sometimes, a post-retrieval assessment for caval injury.

**Figure 4.**
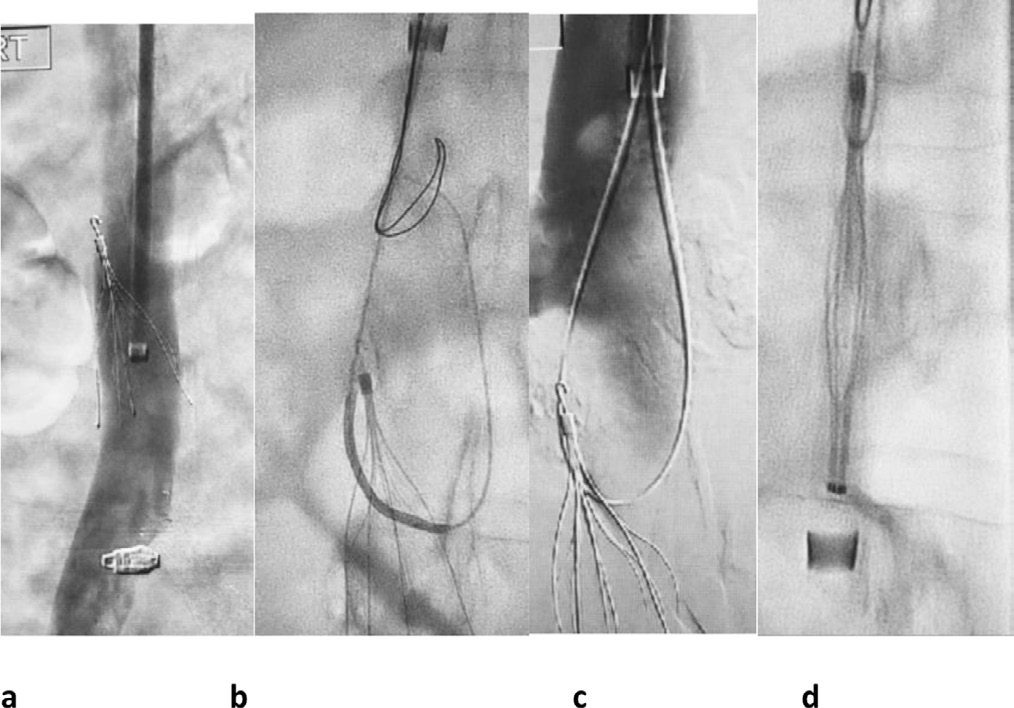
Example of a difficult retrieval. Initial standard retrieval methods had failed as (a) the filter had tilted against the wall of the IVC and endothelialised. (b) Using a long 16 Fr sheath and a Rim catheter (AngioDynamics, New York) , a 300 cm x 0.014 inch pilot wire was looped through the struts of the filter. (c) The 0.014 inch guidewire was then snared using a filter removal loop snare. The guidewire was brought out through the sheath to the skin so that a long loop of guidewire is present from the skin to the tip of the filter. (d) The filter was straightened by pulling both ends of the guidewire and the sheath was advanced over the hook and the filter removed. IVC, inferior vena cava.

**Figure 5.**
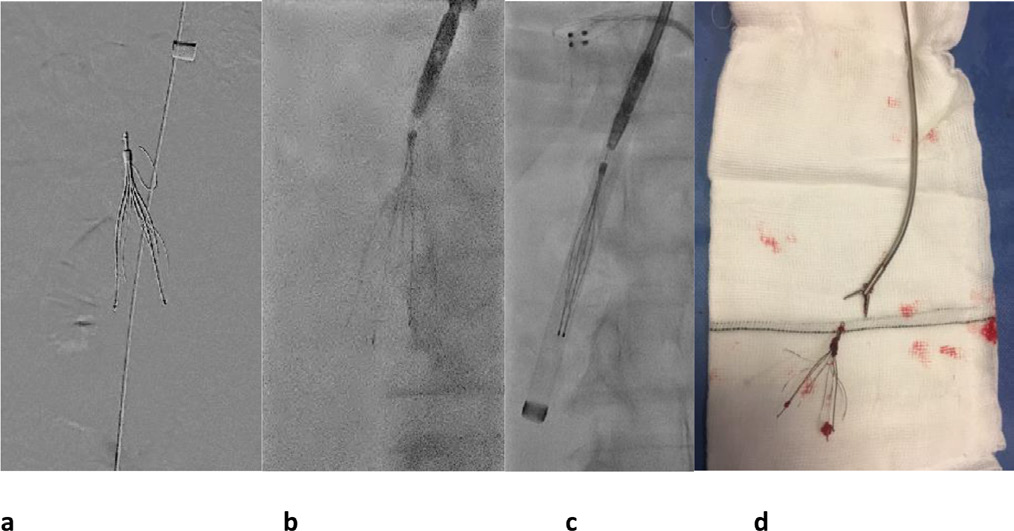
51-year-old lady with unprovoked above knee DVT, saddle pulmonary embolus, and intracranial bleed had an IVC filter inserted. Multiple attempts were made to remove the filter with standard snare techniques, but the hook of the filter was embedded in the anterior wall. (a) Attempts at removing the filter caused an arm strut to bend cranially. (b) The patient had a 16 Fr long sheath placed through which an ENT forceps, which was manually curved before insertion, was manipulated on to the hook at the top of the filter and the hook grasped. (c) While keeping the forceps closed around the hook of the filter, the sheath was manipulated down over the filter and the filter removed. (d) The filter and ENT forceps are shown ex vivo. IVC, inferior vena cava; DVT, deep venous thrombosis.

The SIR suggests removal with advanced retrieval techniques, including bidirectional access, snares, looped guide wires (Figure 4), and high-pressure angioplasty balloons when routine techniques fail if the expertise is available and after evaluating risks and benefits.^8,75^ Complex retrievals include using semi-rigid forceps and a larger 16 or 18 Fr jugular sheath (Figure 5). If all of these measures fail, laser-assisted removal is an option. Complication rates are higher when advanced techniques are used^73^ and require performance in a specialist centre.

## Complications and management

**Figure 6.**
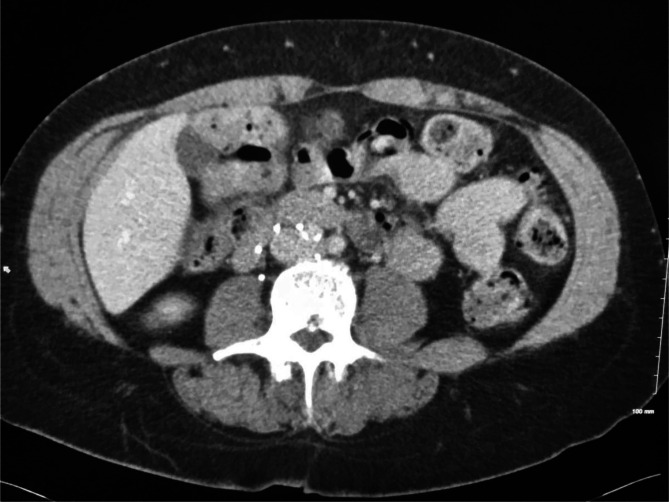
Axial CT demonstrates IVC filter wall penetration of an anchoring strut toward the duodenum on the right. IVC, inferior vena cava.

IVC filter complications can happen in the course of or promptly following placement; however, the majority of complications happen longer than 30 days post-placement when filters are not retrieved.^10^ Initial general procedural complications include bleeding or infection at the venepuncture site, arterial puncture, arteriovenous fistula, and post-procedure thrombosis or haematoma. Early complications specific to filter placement include incomplete opening, filter malpositioning and IVC penetration (Figure 6). Complication rates associated with insertion are between 5 and 23%.^14^

**Figure 7.**
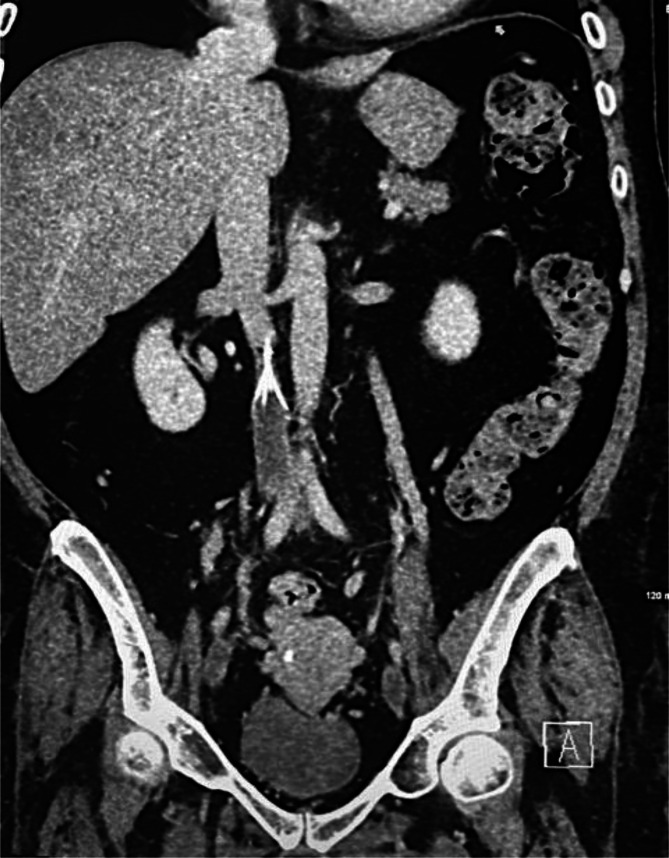
Coronal CT image demonstrates IVC thrombus below the filter. IVC, inferior vena cava.

Later complications are often discovered incidentally and can be seen on cross-sectional imaging^76^ or at the time of attempted retrieval. Unretrieved filters can lead to DVT, migration or embolisation, filter fracture, caval perforation, and filter-related caval thrombosis (Figure 7); all of which usually do not occur within the first 30 days.^10,14,77^ Retrieving filters with evidence of adjacent bowel penetration is safe and technically feasible.^78^

Studies have reported an increased risk of DVT with IVC filters.^79,80^ The risk rate of DVT varies between 5 and 18% and increases the longer the filter remains in place.^10^ However, anticoagulation solely based on filter presence is not recommended except in patients with active malignancy.^8^ Although, anticoagulation, while filters are in place, may reduce long-term filter complications. A retrospective review of 80,697 non-cancer patients hospitalised for acute VTE reported that recipients of IVC filters had a higher 1-year incidence of recurrent DVT (5.4% *vs* 3.7%) than those without filters. The 8-year follow-up from the PREPIC trial reported a hazard ratio of 1.49 (95% CI, 0.99–2.23) for the development of recurrent DVT in filter recipients.^17^

Surgical management over endovascular management may be necessary in some cases.^75,81^ Migration to the heart or lungs can cause cardiac tamponade, chamber perforation, myocarditis, tricuspid valve damage, or death. A meta-analysis reported a rate of 1.3% for filter migration, 90% of which occurred longer than 30 days after insertion.^10^

The SIR reports filter fracture rates between 2 and 10%.^14^ A retrospective series including 363 patients who had Bard Recovery filters found the Kaplan–Meir risk of filter fracture at 5.5 years to be 40%.^15^ Complications reporting IVC perforation rates comprise 20% of overall complications as reported to the Manufacturer and User Facility Device Experience (MAUDE, part of the US Food and Drug Administration)^10^ with similar rates of wall penetration (19%) found in a systematic review including 9002 procedures.^82^ Filter perforation is usually asymptomatic, frequently occurring when the filter is in situ for more extended periods and tilted by greater than 15 degrees.^16^ IVC thrombosis may be due to the thrombogenicity of the filter or a trapped embolus from a distal site. Reported rates of IVC thrombosis are between 2 and 30%,^14^ with one meta-analysis of multiple filter types reporting an overall rate of IVC thrombosis or stenosis of 2.8%.^10^

Table 3 lists common IVCF complications.

**Table 3.** Indications for inferior vena cava filter use from professional societies

Proposed indication	BSH, 2006^83^	AHA, 2011^84^	^85^ESC (2014)/2019^21^	^9,9^ACR, 2019)	ASH, 2019^86^	SIR, 2020^8^	NICE, 2020^87^	ACCP, 2016^1^ 2021^88^
Acute VTE in addition to AC/without CI to AC	Not supported (Grade A, Level 1b)	NR	Not supported (Class III, Level A)	NR	NR	Not supported	Not supported	Not supported (Grade 1b)
VTE and CI to AC	Supported (Grade B, Level III)	Supported (Class I, Level B)	Supported (Class IIa; Level C)	Supported	NR	Supported	May be appropriate	Supported (Grade 1b)
Acute VTE and major complications of AC	NR	Supported (Class I, Level B)	NR	Supported	NR	Supported		NR
Recurrent VTE despite appropriate AC (“failure of AC”)	It may be appropriate after an alternative discussion of AC (Grade c, Level IV)	It may be appropriate (acute PE recurrence) Class IIa. Level C	Supported (Class IIa; Level C)	Supported	NR	Not supported	IMay be appropriate after other options explored	NR
Chronic VTE (*e.g.,* Chronic Thromboembolic Pulmonary Hypertension)	NR	NR	Not supported	May be appropriate if CI to AC	NR	NR	NR	NR
Nondeferrable surgery requiring AC interruption with a recent history of VTE (<1 mo)	May be appropriate (Grade C, Level IV)	NR	NR	NR	NR	NR	NR	NR
DVT/PE undergoing advanced therapies, including thrombolysis/thrombectomy	Not supported (Grade C, Level IV)	Not supported (Class III, Level C)	NR	May be appropriate	NR	May be appropriate	NR	NR
Free-floating iliofemoral or IVC thrombus	Not supported (Grade B, Level III)	NR	Not supported	May be appropriate	NR	NR	NR	NR
Patients with cancer with acute VTE as an adjunct to AC	Not supported	NR	May be appropriate if AC CI	Not supported	NR	NR	NR	NR
Severe cardiopulmonary disease/poor reserve and DVT or PE	NR	May be appropriate (acute PE with poor reserve) (Class IIb, Level C)	Not supported	May be appropriate	NR	May be appropriate	NR	May be appropriate
Primary prophylaxis trauma	NR	NR	NR	May be appropriate	Not supported	Not supported	NR	Not supported (Grade 2C)
Primary prophylaxis bariatric surgery		NR	NR	May be appropriate	Not supported	Not supported	NR	Not supported (Grade 2C)
Primary prophylaxis high-risk orthopaedic surgery	NR	NR	NR	May be appropriate	Not supported	Not supported	NR	Not supported (Grade 2C)

AC, anticoagulation; ACCP, American College of Chest Physicians; ACR, American College of Radiology; AHA, American Heart Association; ASH, American Society of Haematology; BSH, British Committee for Standards in Haematology; CI, Contraindicated; CTEPH, chronic thromboembolic pulmonary hypertension;DVT, deep venous thrombosis; ESC, European Society of Cardiology; NICE, National Institute for Health and Care Excellence; NR, not reported;PE, pulmonary embolism; SIR, Society of Interventional Radiology;VTE, venous thromboembolism.

## Expected direction

The PRESERVE trial, a collaboration between the SIR and the SVS, is the first large-scale prospective clinical study to evaluate the use of IVC filters and related follow-up treatment in the USA.^89^ Observation of the patients will continue for 24 months with the composite primary end point of safety, free from clinically significant embolisation, perforation, occlusion, and DVT. Data from this study will help guide decision-making on IVC filters across multiple disciplines.

Recently, there has been the development of multidisciplinary pulmonary embolism response teams (PERT), which monitor patients acutely in the hospital, followed by outpatient follow-up visits. Hopefully, these teams could reduce unnecessary IVC filter placements and facilitate follow-up in a specific VTE clinic, leading to higher filter retrieval rates.^90^

There are modifiable convertible filters that convert to an open formation that does not filter the IVC after the risk of PE passes. One group inserted a convertible filter (VenaTech^®^ Convertible TM, B. Braun Interventional Systems, Pennsylvania) in 149 patients with VTE. When deemed appropriate, the filter could convert by snaring the filter hook to unlock and open the filter to become a stent incorporated into the caval wall. There was a high conversion rate with a low incidence of adverse effects.^91^ Bioconvertible filters can convert to an open position independently following insertion.^92^ There are central venous catheters with deployable IVC filters (filter on a stick, Angel^®^ Catheter, Mermaid Medical, Denmark) available for clinical use, which can be placed at the bedside via the femoral vein for critically ill patients.^93^

## Conclusion

Significant uncertainty remains regarding managing patients with VTE, particularly in limited disease and special patient populations. Professional society guidelines vary in their recommendations for expanded indications. A literature review suggests that IVC filters are not definitively beneficial beyond the classic indication. IVC filters have higher complication rates, with filters failing to be retrieved, but overall complication rates remain low. Multiple permanent and retrievable/optional filter devices are available for use. More recently, device options have expanded with the development of convertible, bioconvertible, and central venous catheter/IVC filter combination devices.

Further prospective studies are needed to quantify the clinical benefits and risks of IVC filter placement in subsets of patients, including, *e.g.* those with VTE recurrence despite therapeutic anticoagulation, those receiving thrombolytic therapy, or those involved in trauma or major surgery. Additional research is needed to optimise patient care for this patient population. We should collect data regarding the short- and long-term clinical and cost-effectiveness associated with IVC filter insertion and appropriate retrieval. Researchers should assess the impact of pre-procedural imaging such as CT *vs* no CT before filter retrieval on procedure success, duration, and costs. High-quality evidence in large prospective randomised trials and registries is needed to provide more robust evidence.
